# Multiphasic movement and step-selection patterns of dispersed tigers in the central Indian landscape

**DOI:** 10.1371/journal.pone.0309517

**Published:** 2024-10-23

**Authors:** Supratim Dutta, Ramesh Krishnamurthy

**Affiliations:** 1 Wildlife Institute of India, Dehradun, Uttarakhand, India; 2 Faculty of Forestry, University of British Columbia, Vancouver, Canada; University of Insubria: Universita degli Studi dell’Insubria, ITALY

## Abstract

Large carnivores play a crucial role in the ecosystem, though their conservation needs a landscape-level approach due to their wide range of habitats and dispersal events. The study of tigers in a human-dominated landscape matrix and their adaptation and adjustment of movement behaviours during the dispersal phase is essential for long-term conservation planning and management policy. We studied the dispersal event of five VHF/GPS collared individuals during 2009–2020. We investigated movement parameters (step length), and the effects of anthropogenic pressures (distance from village), distance from water and vegetation cover, on behavioural phase under a Hidden Markov Model framework. We also tested the effects of distance from village, vegetation cover, and habitat types on animal movement using an integrated Step Selection Function framework. The mean step length (405.44±10.63 m/hr) varied widely by different time of day. Displacement was high during the night (665.28±21.36 m/hr) compared to day (434.16±17.37 m/hr). Tigers moved fast (872.7m; 95% CI 839.1–906.3m) with longer step length and a directional turning angle in non-forested areas (i.e. the human-dominated landscape), likely to avoid conflict with humans. Individuals distinctly exhibited two behavioural states: encamping (~32% of the time) and travelling (~68% of the time). Further, they avoided the human-dominated landscape and mostly remained in and forested areas, especially during nighttime. Our study is the first attempt to understand behavioural transition of dispersal tigers and their habitat selection. Lesser anthropogenic disturbance and high vegetation cover positively influenced the tiger dispersal, while water availability did not affect their state transitional probability. Additionally, dispersers showed high affinity towards forested land during nighttime for travelling.The findings of this study show the importance of functional corridors and stepping stones (mostly encamping areas), and also provide baseline knowledge for integrated landscape management planning and policymaking for the long-term survival of tigers in metapopulation framework.

## Introduction

Conservation of large carnivores is a major challenge across the globe. In the Indian landscape, major conservation programmes depend upon various political, financial, socio-economical and mythological aspects that can complicate conservation efforts. Tiger populations in small Protected Areas (PA) are vulnerable to extinction events due to demographic, environmental and anthropogenic factors [1, 2]. The Central Indian landscape is one of the most dense tiger populations in the world [3]; simultaneously, this landscape is highly fragmented [4, 5]. Due to extensive poaching, the native population of Panna became extinct in 2009 [6, 7]. After successful reintroduction in 2009, the tiger population reached carrying capacity, and animals are now moving outside the PA. Therefore, landscape-level connectivity is a severe concern in the Greater Panna landscape, involving multiple PAs, and requires an integrated landscape-level management approach with demarcation of functional or potential corridors to ensure the safe passage of the dispersed animals [8]. As the dispersal event is one of the most important life-history traits and is governed by several biological and environmental factors [9], a strong policy towards connectivity management taking into account interface aspects is required to secure the movement of dispersal animals.

Movement is one of the most fundamental and crucial abilities for the survival of large carnivores, allowing animals to find essential resources (e.g. food, shelter, mates); animals need a balance of all these resources to maximize their fitness [10, 11]. Large carnivores need a comparatively larger area to fulfil their life requisites [12]; this needs sometimes forces them to disperse from their natal place to multi-use lands, especially those outside the PA [13]. Therefore, the survival of large carnivores depends on their adaptability to the human-modified landscape. Individuals may develop flexible behavioural strategies to minimize the spatio-temporal overlap with humans and strategically use anthropogenic resources by altering movement behaviour and habitat use [14–16]. This can be achieved by increasing speed to avoid human distrubance [17], minimizing daytime activity [18], and increasing nocturnal activity [19, 20].

Animal movement is dependent on internal state, motion capacity, and navigation capacity; it is influenced by both internal and external biotic and abiotic elements that interact with the individual [21]. The impacts of these factors may be reflected in animal’s movement behaviour [22, 23]. The recent development of GPS technology provides more precise locations in animal tracking [24, 25]. The Hidden Markov Model (HMM) can be used to characterise animal movement from a finite number of hidden behaviour states [26, 27]. The behavioural state process is known as Markov chain, and the state at the subsequent time step depends only on the present state. The different behavioural states are characterized by step length and turning angle [28]. Similarly, habitat selection reflects the behavioural-based drive to compete and fulfil context-dependent requirements [29]. Habitat or resource selection is highly governed by the animal’s behaviour [30–34].

With the advancement of technology and analytical tools [35], this fine-scale movement data allows for the incorporation of explicit behaviour into habitat selection studies [36]. To assess fine-scale habitat selection from telemetry data, step-selection function (SSF) is a powerful tool [37–39] to estimate the relative probability of selecting a certain habitat (by the animal) versus alternative available habitats across the landscape. The integrated step-selection function (*i*SSF) incorporates an animal’s movement in a defined available area; additionally, *i*SSF can account for the temporal variability in environmental conditions as the animal navigates in the landscape [40]. To optimize fitness, animals sometimes need to adjust their habitat selection [41, 42] by avoiding human-modified features [32, 43] and can display a temporal shift in activity.

Dispersal of tigers through the human-dominated landscape is common, but most studies have been restricted to trajectory-based dispersal [17, 44, 45] and habitat preference during movement [4]. Therefore, it creates a knowledge gap that how does the dispersers change the behavioural patterns and select habitat during the dispersal. Our study is unique in that we evaluated i) the changes in step length of tiger during day and night, ii) the behavioural states of the animal during the dispersal event and iii) the effects of human disturbance and forest on their behaviour and habitat selection. We hypothesized that the dispersing animals would move faster at night compared to daytime and would alter their behavioural states based on the habitat and that dispersers preferred to travel more than encamping. We further hypothesized that tigers preferred forested area during nighttime.

## Materials and methods

### Study area

**Fig 1:**
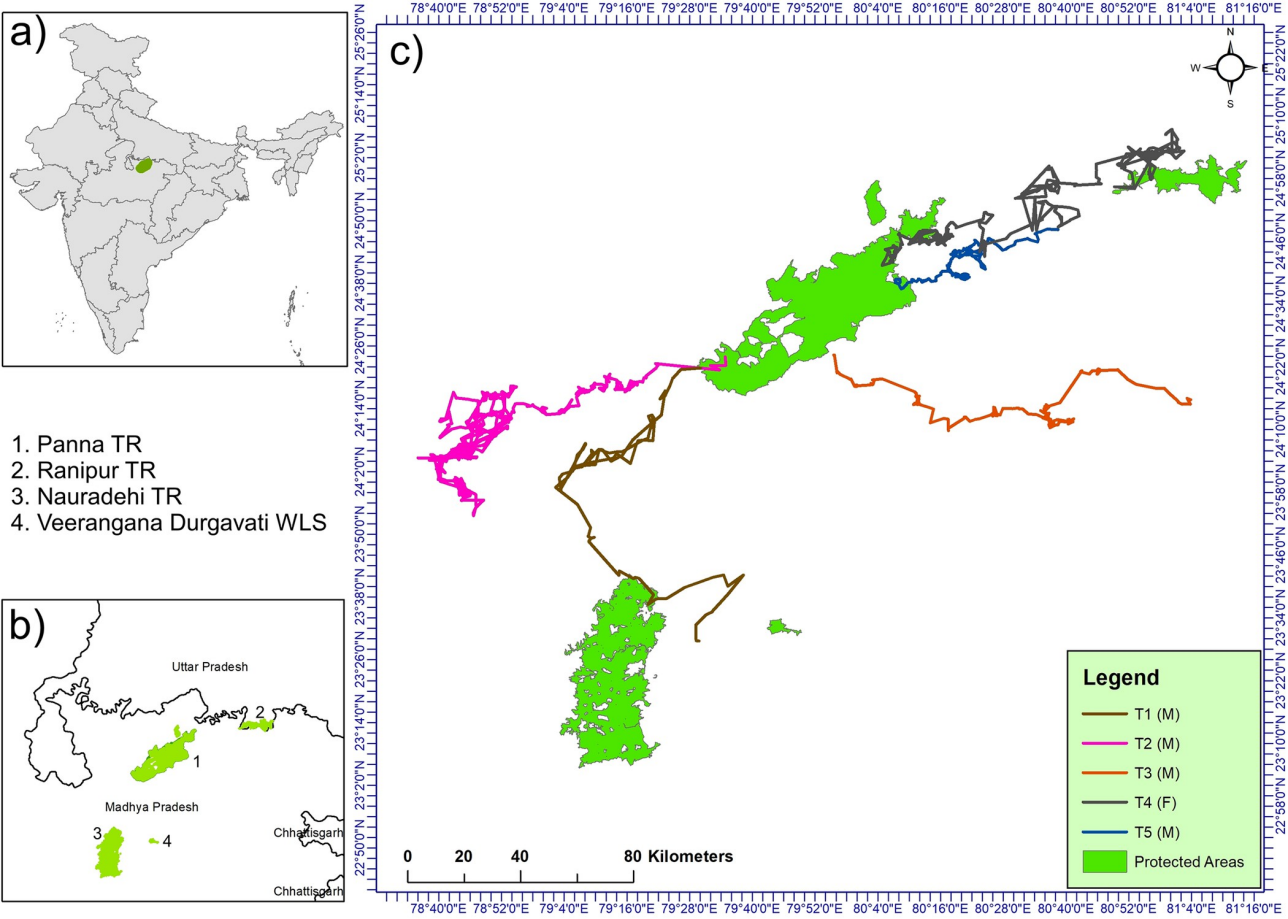
Map showing the position of the study area: a) India, b) the states of Madhya Pradesh and Uttar Pradesh, and c) position of four different protected areas in the landscape. The coloured lines are representing the movement pathway of tigers.

The study was conducted in the northeastern part of the central Indian highlands (Vindhya Range). The landscape is spread over five districts (Panna, Chhatarpur, Damoh, Sagar and Satna) within the state of Madhya Pradesh. Panna Tiger Reserve (PTR) is at the focal point of the landscape. The landscape contains three other PAs (Ranipur Tiger Reserve, Nauradehi Wildlife Sanctuary, and Veerangana Durgavati Sanctuary; Fig 1). The Ken and Bearma rivers are major water sources in this landscape, both of which run from south to north. The elevation ranges from 580 to 230 masl, while the average annual precipitation is ~1100 mm. The sub-tropical dry climate shows three distinct seasons: a) mostly hot, dry summer, b) comparatively humid monsoon, and c) dry winter; the temperature varies from 5-45°C. The study area is categorized as tropical dry-deciduous forest [46]. Champion and Seth broadly classified the area as group 5, ‘Tropical Dry Deciduous Forest’ [47]. Apart from Tiger (*Panthera tigris*), the landscape contains substantial population of Leopard (*Panthera pardus*), Striped Hyena (*Hyaena hyaena*), Indian Wolf (*Canis lupus*) and other carnivores like Jackal (*Canis aureus*), Asiatic Wild Dog (*Cuon alpinus*), and Indian Fox (*Vulpes bengalensis*). Similarly, the forested area harbours a viable population of Chital (*Axis axis*), Sambar (*Rusa unicolor*), Nilgai (*Boselaphus tragocamelus*), Chinkara (*Gazella bennettii*), Chousingha (*Tetracerus quadricornis*), Blackbuck (*Antilope cervicapra*), and Wild Boar (*Sus scrofa*). The landscape is highly fragmented with cities, roads, rural settlements, industrial areas and croplands.

### Data collection

#### Radio telemetry

From March 2009 to December 2020, we captured 28 individuals (9 males and 19 females) under the long-term projects entitled “Tiger Reintroduction and Recovery Programme in Panna Tiger Reserve, Madhya Pradesh” and “Development of Landscape Management Plan and Monitoring Strategy with reference to Ken–Betwa River Link Project in Panna Tiger Reserve, Madhya Pradesh.” Animals were captured and collared under the permit of the Madhya Pradesh Forest Department (MPFD Letter No./Exp./2009/1205 dated 31/8/09 and WII/KR/PROJECT/PLMP/2017-18/F(1)) following the capture rules and regulations of the Wildlife Protection Act, 1972 section 11 (1A). The target animals were tracked and immobilized using a ‘Hellabrunn mixture’ (125 mg xylazine + 100 mg ketamine/ml) [48] injected through a Tele-inject projector (Model 4V.31) by a professional veterinarian. Tigers were collared with VHF/GPS/UHF collars (African Wildlife Tracking^®^ Inc and Vetronic Aerospace^®^) and Very High-Frequency transmitters (Telonics^®^ Inc). All collared tigers were monitored intensively with UHF and satellite tools. Here we used the telemetry data of five dispersed tigers (four males and one female, only during dispersal period; S1 Table and S1 Fig in S1 File), those who moved outside to the park. We did not include the non-collared disperse tigers in this study.

### Analytical methods

#### Location of animals

We obtained the GPS data hourly, though missing GPS fixes are common in telemetry-based studies [49]. Most behavioural studies require continuous time-series data [50–52]. To overcome this problem, we predicted the missing locations using the ‘crawl’ package in R [53, 54].

#### Displacement

We calculated the mean displacement (step length) using the ‘adehabitatLT’ package in R [54, 55]. To understand the movement behaviour on a more fine-scale during the dispersal phase, we categorized the time into two segments, day and night. Further, we performed Mann-Whitney U test to check the significance difference in step length in different temporal frame.

#### Multi-phasic behavior

We used the HMM [27, 56] approach to segment the individuals’ movement trajectories into behavioural states based on the consecutive step length (linear distance between two successive GPS fixes) and turning angles (the angle between two successive steps). We applied the Gamma distribution and Wrapped Cauchy distribution to model the step length and turning angles, respectively. Since our study focused on the movement of dispersed tigers, we classified data into two behavioural phases: ‘encamping’ and ‘travelling [51].’ We assigned the initial mean gamma step length of 100m (minimal displacement), standard deviation of 50m, and zeromass parameter of 0.01 with 0.3 turning angle concentration (corresponding to turning back) for the resting phase. For the travelling phase, 700m mean gamma step length (larger displacement) was assigned with 1000m standard deviation and a zeromass parameter of 0.05 with 0.7 turning angle (directional) [28]. We projected the transition probabilities among behavioural states; we applied the Viterbi algorithm to investigate the state probability of the animal during dispersal and for each consecutive step to understand the animal’s behavioural state based on the HMM output [57]. We performed the HMM analysis in R using the package ‘momentuHMM’ [51, 54]. Additionally, anthropogenic factors, such as proximity to villages, adversely affect animal movement [4]. Consequently, we incorporated distance to villages, distance to water, and normalized difference vegetation index (NDVI) as covariates to investigate their influence on the behavioral phases of tigers. The build-up class data was sourced from Copernicus (https://lcviewer.vito.be/2019) using a 75% threshold level to identify village and settlement areas. For each location, we calculated the linear distance to the edge of the nearest settlement. NDVI data was obtained from the Google Earth Engine cloud computing service using the image number ’LANDSAT/LE07/C02/T1’ (2009 and 2012) and ’LANDSAT/LC08/C02/T1_TOA’ (2014, 2016, 2021; https://code.earthengine.google.com/). Similarly, spatial layers of water were extracted from the Forest Survey of India, 2014 (www.fsi.nic.in) data. The distance to water layer was subsequently prepared by applying the euclidean distance from the nearest water source. All spatial layers were reclassified and resampled at a 100m scale in R using the ’raster’ package [58].

#### Understanding the effects of habitat and heterogenous landscape in movement

We used NDVI and distance to the village as covariates along with forest types, such as non-forest, open forest, and forest. Forest type data were obtained from the Forest Survey of India, 2014 (www.fsi.nic.in). The data comprised six classes: dense forest, moderately dense forest, open forest, shrubland, non-forest and water. This landscape contains minimal dense forest area; thus, focusing on the species ecology, we merged the dense and moderately dense forests into a single class ‘forest.’ Similarly, open forest and shrubland were merged into the ‘open forest’ class. The non-forest area represents human habitation and agricultural fields in the landscape. All covariates were standardized within the dataset to facilitate coefficient interpretation [59].

SSF is a type of resource selection function (RSF) [60]. We used the *i*SSF, an extension of SSF, to quantify habitat selection. *i*SSF allows for modelling of the available movement path and the simulating points generated by random steps [61, 62] to model the habitat selection process and includes the influence of habitat covariates during movement [63]. We generated 15 random points for each dataset point by generating random steps and turning angles projected from the previous point. We used gamma distribution for step length and von Mises distribution for turn angle. The *i*SSF analysis was performed using the ‘amt’ package [64], and the coefficients were estimated by conditional logistic regression using the ‘survival’ package in R [54, 62]. We developed three candidate models for *i*SSF (S2 Table in S1 File). Furthermore, to estimate the effects of habitat covariates, we calculated the relative selection strength (RSS) across the range of two focal habitat covariates [65].

## Results

Since the reintroduction in 2009, 37 tigers have dispersed from PTR. A total of 7,076 data points were obtained from five dispersed collared individuals (S2 Fig in S1 File). We observed that most of the dispersal events took place during the monsoon and winter seasons (August to March), varying from one to three months based on the routes and distances.

### Displacement

**Fig 2:**
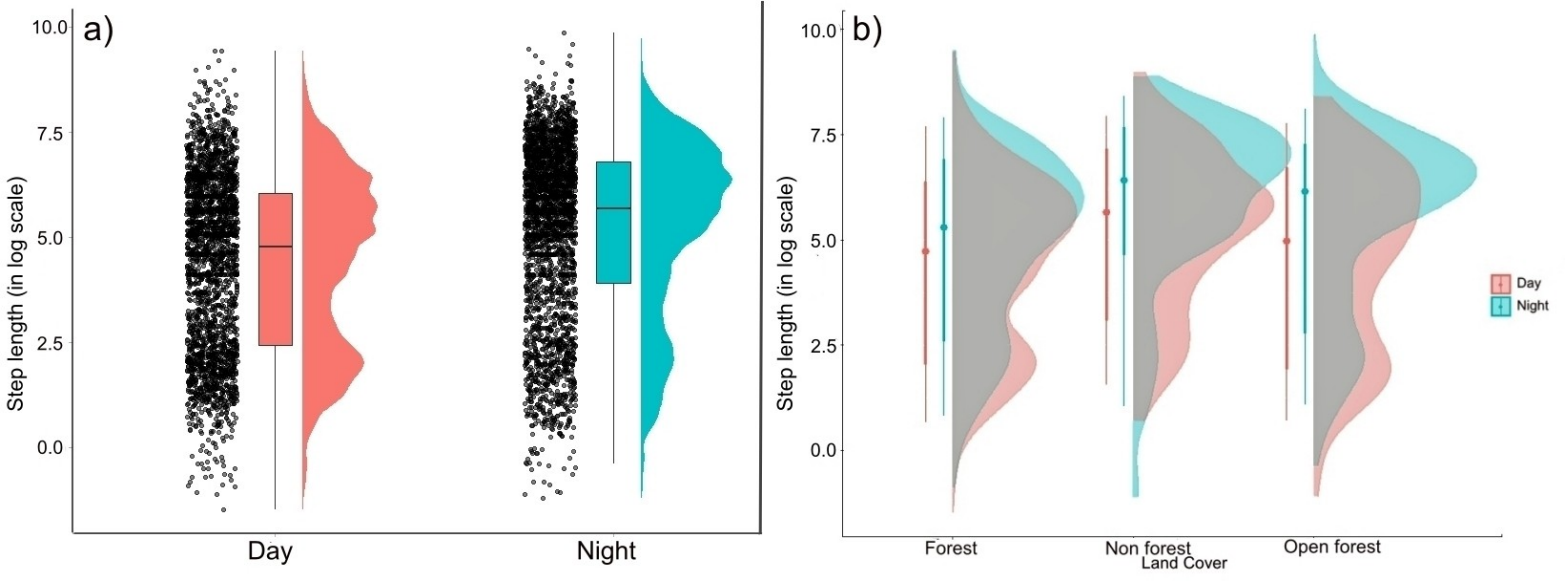
Plot showing a) the change in mean displacement (step length) during day and night and, b) the variation of displacement during day and night across different forest types during the dispersal events.

Outside the PA, the mean (±SE) displacement was 405.44±10.63 m/hr, though it varied widely by time of day (S3 Fig in S1 File). We observed the highest mean displacement during the night (665.28±21.36 m/hr), whereas the mean displacement during the day was 434.16±17.37 m/hr (Fig 2A). However, these were significantly not different (Z 0.25, p value 0.80).

#### Multi-phasic behavior

**Fig 3:**
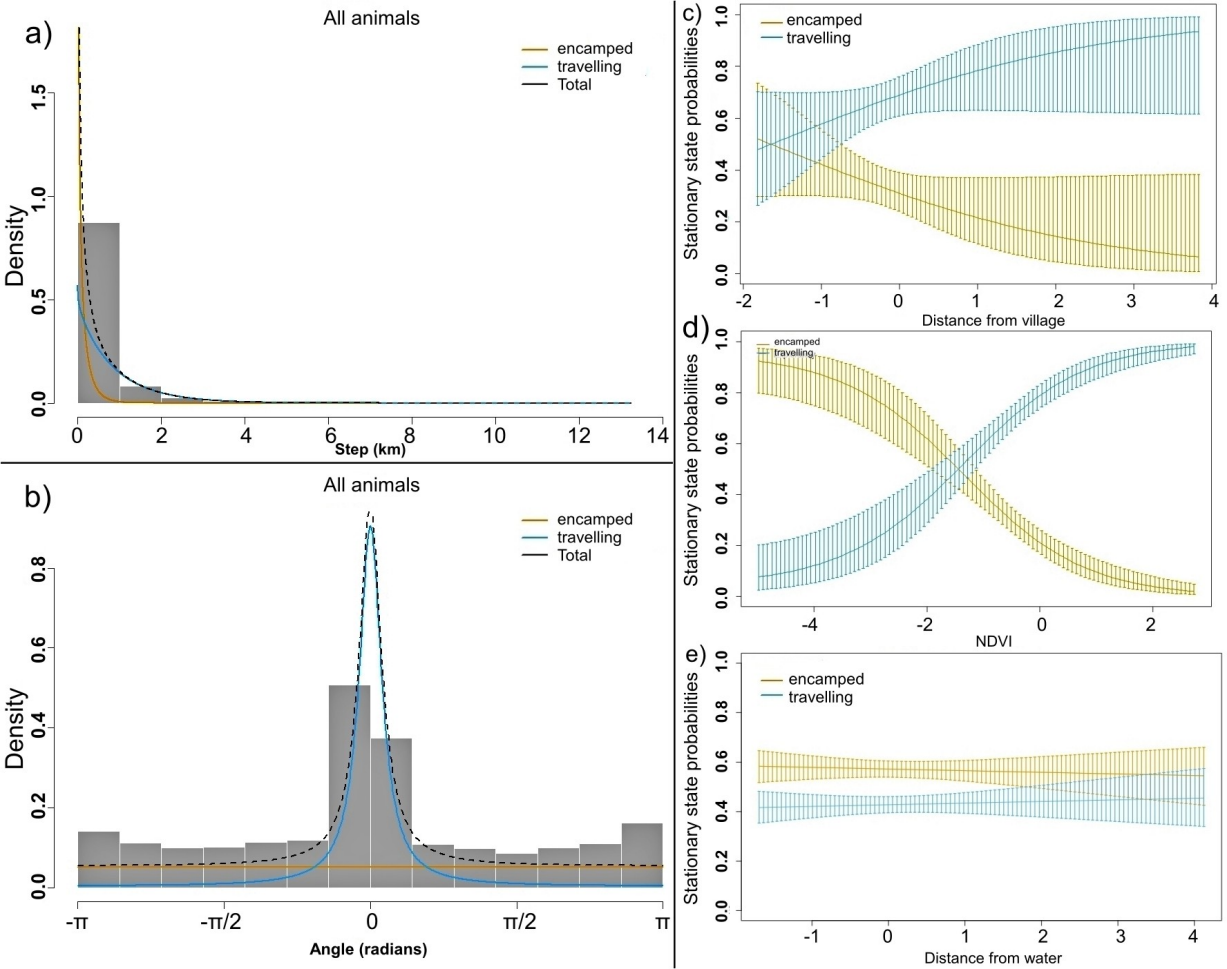
Histograms of observed a) step length and b) turning angle of dispersed tigers. Coloured lines indicate the estimated densities by state, and the dotted black line is their sum. Stationary state probabilities of dispersed tigers as a function of c) distance from the village, and d) normalized difference vegetation index (NDVI), and e) distance from water. Vertical coloured lines represent the point-wise 95% confidence intervals.

The HMM distinctly identified two different behavioural states with different extrinsic covariates depending on transition probabilities. The two-state models predicted the tiger movement precisely; the encamping state had a short step length with undirected turning angle, while the travelling state was characterized by a longer step length (fast and directed movement) with a concentrated turning angle (Fig 3A, 3B). Based on the behavioural states, the mean gamma step length was 107.2m (95% CI 96.7–117.6) for the encamping state and 872.7m (95% CI 839.1–906.3) for travelling state. The Viterbi state sequence indicated that dispersed tigers were more likely to be travelling than encamping, spending 32% of their activity in encamping and 68% in travelling. Furthermore, with an increasing linear distance from the village, tigers were more likely to shift to the travelling phase from the encamping phase (Fig 3C; S4 Fig in S1 File). Tigers moved faster in lands with a higher NDVI during the dispersal event, indicating a preference to travel faster in forested lands (Fig 3D; S5 Fig in S1 File). However, we did not observed any significant state transition of dispersers in response to water availability (Fig 3E; S6 Fig in S1 File). When animals were near to the water, they exhibited marginally higher probability of encamping over travelling.

#### Understanding the effects of habitat and heterogeneous landscape on movement

**Fig 4:**
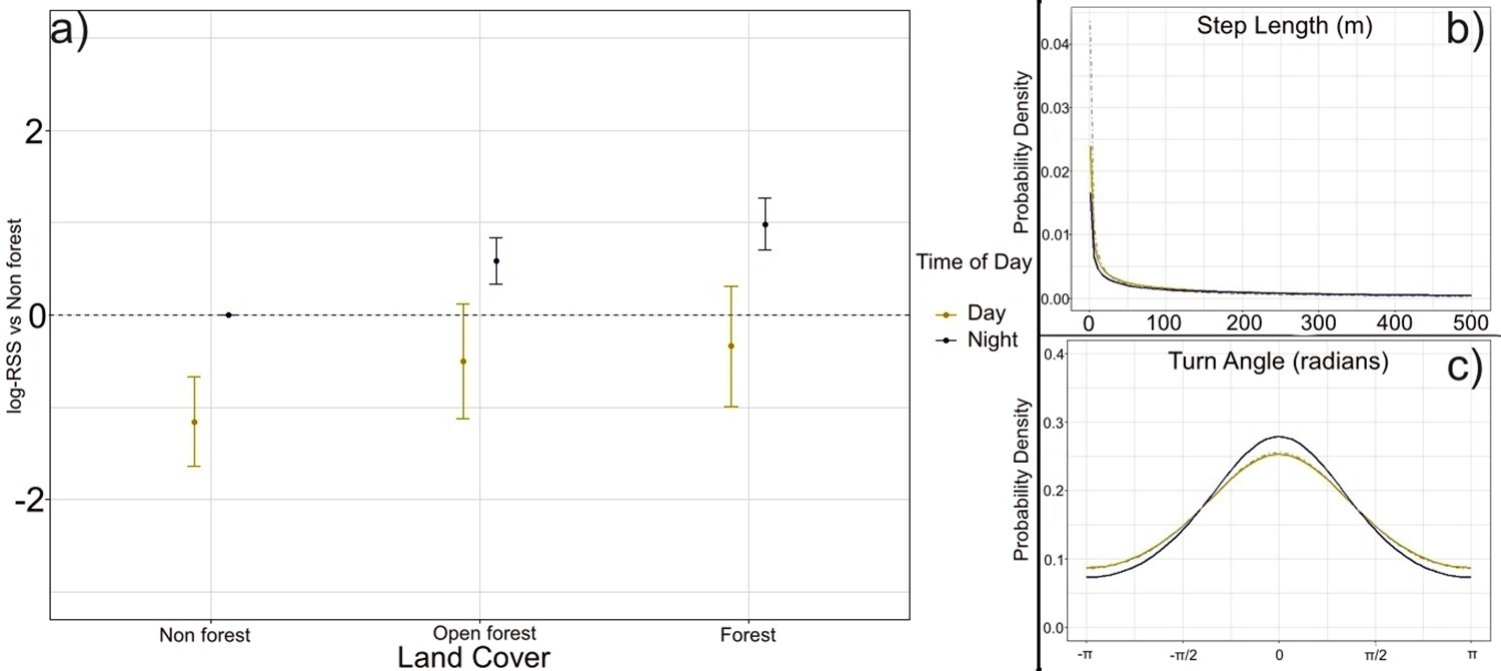
a) Effects of land cover types on dispersed tiger movement, b) change in step length and c) turning angle during the day (yellow) and night (blue) during the dispersal events.

We evaluated three different candidate models (S2 Table in S1 File). NDVI and distance to the village had a strong influence on animal movement and habitat selection. Dispersed tigers moved more slowly with shorter step lengths and high repetitive turning angles during the daytime, while step lengths were a few folds larger with directional turning angles indicating travel across a greater area during the nighttime (Fig 4B, 4C). We found that animals strongly preferred areas with high forest cover and avoid anthropogenic disturbance. During the nighttime tigers preferred forested areas over open forest and non-forest (Fig 4A). Tigers strongly avoided non-forested areas during the day, followed by open forests and forests (Fig 4A; Table 1). Relatively longer step lengths were observed in non-forested or human-dominated areas (day: 690.2±85.8 m/hr, night: 1085.6±79.9 m/hr), indicating that dispersed individuals tried to move faster in the non-forested patches, possibly avoiding spatio-temporal conflict with humans, especially in the agricultural fields and near the villages (Fig 2B). Step lengths were much shorter in forested areas (day: 387.2±31.2 m/hr, night: 537.9±35.8 m/hr), away from the human-dominated area. Longer step lengths were observed in all three different habitat types during the nighttime (open forest: 763.5± 70.4 m/hr) compared to daytime (open forest: 484.9±43.17 m/hr). This also signifies animals moved slower during the daytime. Additionally, we tested the significance of the difference between day and nighttime displacement across different forest types. Our analysis revealed a significant difference in displacement in all three forest types (non forest: Z-3.47, p value = <0.001; open forest: Z-4.14, p value = <0.001; forest: Z4.58, p value = <0.001).

**Table 1:** Candidate model tested in step-selection function analysis.

Factor	Coefficient	SE	P value
NDVI	2.141	1.11	0.053 .
Distance to village	2.007e-04	4.241e-05	2.23e-06***
Forest area during day	0.816	0.190	1.81e-05***
Open forest area during day	0.649	0.172	0.000165***
Forest area during night	0.980	0.144	9.63e-12 ***
Open forest area during night	0.586	0.128	4.87e-06 ***
Turning angle	-0.011	0.044	0.792762
Log (Step length) during day	0.186	0.014	< 2e-16 ***
Log (Step length) during night	0.230	0.014	< 2e-16 ***
Step length during day	-3.520e-04	5.908e-05	2.54e-09 ***
Step length during night	-8.229e-05	3.765e-05	0.028855 *
Turning angle during night	0.137	0.006	0.023945 **

Signif. codes

0 ‘***’ 0.001

‘**’ 0.01

‘*’ 0.05 ‘.’ 0.1 ‘ ‘ 1

**Fig 5:**
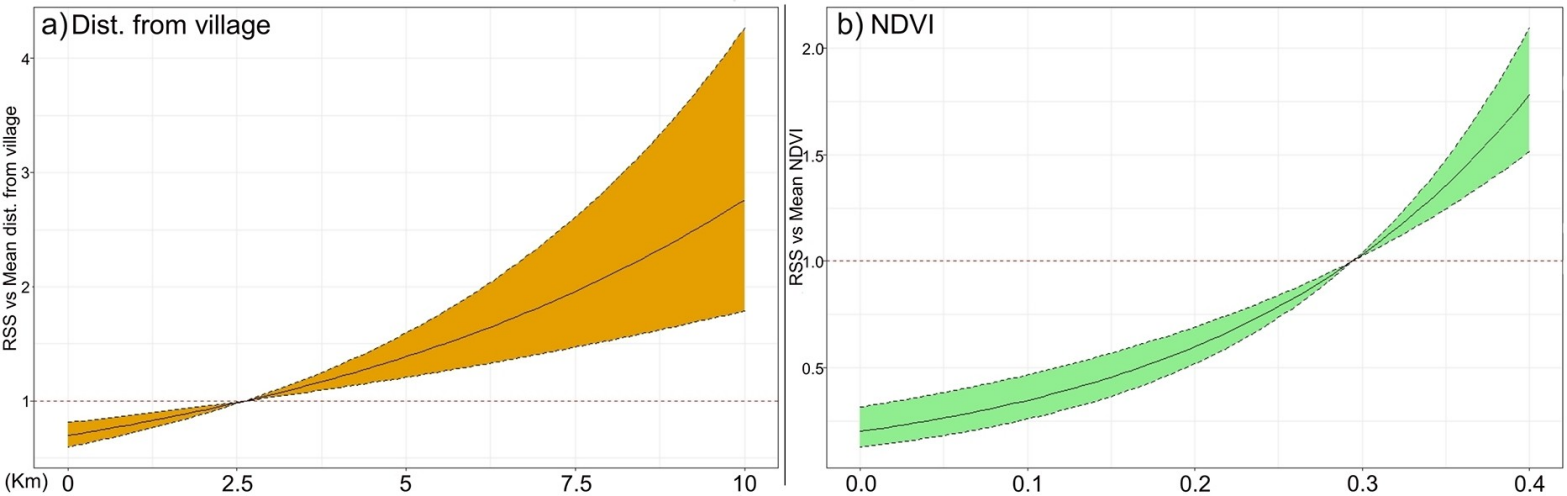
Relative selection strength (RSS) of a) distance to the village and b) normalized difference vegetation index (NDVI) by the tiger. Coloured areas surrounding the curves represent 95% confidence intervals.

Our results indicate that dispersers are likely to select a habitat far from village sites and in areas with higher NDVI values. Tigers preferred a habitat 1.8 (CI: 1.42–2.42) times more in an area 5km away from the village compared to an area 500m from the village site at NDVI value of 0 (Fig 5A). Additionally, an animal was about 5.13 (CI: 3.24–8.14) times more likely to step into a habitat with NDVI value of 0.3 compared to NDVI value of 0 at a 5km distance from the village site (Fig 5B).

## Discussion

This study comprehensively demonstrates the first-ever detailed movement-based behavioural insights of tigers in a highly human-dense areas and fragmented landscape. To fulfil their needs, animals may select different habitats with different behavioural strategies during their dispersal phase. Here, we show that dispersed animals exhibit different behaviours in anthropogenic/agricultural areas compared to forested areas. We found tigers moved at higher speeds during night compared to the day, potentially to cover larger distances in a fragmented landscape; similar studies on carnivores, such as Tigers, African Lions and Cougars, in human-dominated landscapes show that the animals exhibit higher speeds while travelling through fragmented areas to reduce travel duration in multi-use lands [17, 66–68]. Earlier studies on tigers in the central Indian landscape have reported shorter step lengths compared to our findings [17]. This discrepancy suggests that the landscape in our study area is more fragmented and poses greater challenges to tiger movement, forcing them to move faster to avoid human-dominated areas.

We found strong evidence of different stationary state probabilities under the HMM framework and their associated time/activity budgets with different movement patterns, as have been described elsewhere [9, 69, 70]. Tigers allotted more of their activity budget for travelling (~68%) compared to encamping (~32%). Dispersed tigers spent most of their time in a highly mobile mode (travelling state: longer step length and directed movement), as they do not need to defend and maintain their territory. Moreover, dispersers more often meet their foraging needs in high-risk lands (near the village) by engaging in depredation of domestic animals or by scavenging [33, 69, 71]. This situation forces the dispersers to adopt a less mobile strategy to capture and handle the prey to fulfil their energy requirements. Our findings show that tigers engaged in resting behaviour near the villages and in lower NDVI areas. Tigers likely restricted their movement when near the village and in lower NDVI zones to avoid unnecessary human-wildlife conflict [44]. Water is recognized as a crucial resource for carnivores [72]. However, in our study, dispersers’ movements were not dependent on water, as most dispersal events occurred during the monsoon and post-monsoon periods. The ample availability of water during these times did not appear to limit tiger movement. Environmental factors strongly influence animal movement behaviour; in our study, dispersers switched their state probability from encamping to travelling state in forested area during the night [73]. The crepuscular and nocturnal behaviour of dispersed tigers [17, 73] provides an extra advantage in that they can travel longer distances (highly mobile state) in colder and darker times. These diel activities and temporally changing behavioural patterns might be explained by thermoregulatory techniques of the carnivores [74], along with making behavioural adaptations to decrease the chance of being detected by competitors or threats, such as humans [75].

In our study, the use-availability framework (*i*SSF) substantially impacted the estimation of habitat selection coefficients in previously defined behavioural states; therefore, recorded values for step length and turning angles were considerably different in this study. Studies suggest that land use variables such as forest type, NDVI, and village matrix would be the critical factors for predicting tiger movement across the landscape [76, 77]. The *i*SSF approach clearly distinguished the dispersers’ movement behaviour in different habitat types. We found land cover (non-forest, open forest, and forest) is one of the essential factors in tiger movement ecology, and animals avoided the agriculture-village matrix, this same result was also shown by Krishnamurthy et al. (2016) [4]. Dispersers strongly avoided non-forested area during the day-time, and similar behaviour has also been found in other carnivores, such as the African Lion [52, 78]. If resources are only available in high-risk areas (i.e. near the village), dispersers modify their behaviour and are likely to forage during low-risk times (mostly during the night) [52]. We also observed distinct movement patterns during day and night. Carnivores generally avoid daytime movement to reduce the risk of conflict with humans, a behaviour documented in other carnivore studies [52]. Our results support this hypothesis, showing similar movement patterns during dispersal. Significant changes in step length at night compared to the day across all forest types indicate that dispersers restricted their daytime movement and covered longer distances at night by increasing their speed. The strong relative habitat selection of areas with higher NDVI and longer distance from the village shows the importance of small forest patches in a human-dominated matrix. Our findings highlight the need to preserve continuous dispersal corridors in this landscape. Building coexistence landscapes should aim to facilitate the geographical and temporal separation of human structures and activities from big carnivore breeding and dispersion habitats [79].

Our study focused on the classification of tracks into different movement states; therefore, we modelled fewer states with precise biological interpretation [80], based on prior knowledge of carnivores [9, 34, 81]. Furthermore, our sample size of individual tigers was small and male-biased; therefore, we recommend further telemetry study of dispersing tigers to accurately map and manage functional corridors. Further, collaring of dispersed tigers may also minimize the chance of direct human-wildlife conflict and prevent related deaths. Overall, the study offers site specific insights and a general framework for better understanding and managing connectivity conservation.

### Conservation implications

Conservation efforts associated with preventing forest degradation and illegal encroachment should be the top priority, ensuring functional connectivity with other PAs [5, 82]. During the dispersal, the encamping zones of tiger can be considered as stepping stones [83]; a small habitatble patch. Stepping stones are crucial for maintaining connectivity, that facilitate wildlife movement across fragmented landscapes [84]. It acts as intermediate habitats, allowing animals to safely traverse between larger habitat areas, ensuring gene flow and reducing the risk of population isolation [85]. In the form of ’Other Effective Area-Based Conservation Measures’ (OECMs), stepping stones can significantly contribute to wildlife conservation outside the protected areas [86, 87]. By integrating stepping stones into long-term conservation planning, an ecologically functional networks can be created, that can support species persistence in human-dominated landscapes. This approach ensures a more comprehensive and resilient strategy for landscape-level conservation.

## Conclusion

Our study is the first comprehensive attempt to evaluate the behavioural phases and habitat selection of large carnivores during dispersal in the central Indian landscape. Hidden Markov Models (HMMs) can infer the latent states underlying observed animal movement data. This method allows for the characterization of movement patterns specific to each hidden state, such as speed, directionality, and step length. Additionally, HMMs effectively capture the temporal dynamics of animal movement, modeling how the probability of being in a particular state changes over time and how these states influence movement patterns [27, 28]. Our findings show how dispersed tigers navigate a human-dominated landscape, particularly outside protected areas. Tigers adjusted their behavioural states during dispersal to adapt to the specific challenges of the landscape, utilizing forested areas and traveling faster at night by increasing both speed and step length. Movement was significantly influenced by disturbance (distance to the village) and vegetation (NDVI), but not heavily reliant on water due to seasonality.

Unlike Resource Selection Functions (RSFs), Integrated Step Selection Functions (iSSFs) consider habitat selection with movement behaviour, providing a dynamic understanding of animal movements [63]. RSFs select random points to represent available habitat [88], which may lead to misleading results, while iSSFs explicitly account for the temporal sequence of locations, allowing for the analysis of movement patterns and decision-making processes at each step. In our study, movement was also influenced by different forest cover types and times of day (diel period). The high affinity of animals for forested areas underscores the importance of small habitable patches [83]. The effectiveness of stepping stones and corridors ensures functional connectivity within a metapopulation framework, contributing to the long-term survival of this majestic species [84, 89].

## Supporting information

S1 File (DOCX)
