# Supplementary material for: Multiphasic movement and step-selection patterns of dispersed tigers in the central Indian landscape
Source: PLoS One. 2024 Oct 23;19(10):e0309517. doi: 10.1371/journal.pone.0309517 (PMC11498731; doi:10.1371/journal.pone.0309517)
Supplement: S1 File — (DOCX) [file pone.0309517.s001.docx]

**Supporting information: 1**

**Title: Multiphasic movement and Step-selection patterns of dispersed Tigers in Central Indian landscape**

Supratim Dutta^1^, Ramesh Krishnamurthy^1,2*^

^1^ Wildlife Institute of India, Dehradun, Uttarakhand, India

^2^ Faculty of Forestry, University of British Columbia, Vancouver, Canada

*Corresponding author: ramesh@wii.gov.in

**S1 Table:** Individual-wise detail of each disperse tigers.

| **Sl No.** | **Tiger ID** | **Sex** | **Age during dispersal** | **Outside the PA** | | | | | | **No. of Fixes** | **Fate** |
| --- | --- | --- | --- | --- | --- | --- | --- | --- | --- | --- | --- |
|  |  |  |  | **Start Date** | **Start Coord** | | **End Date** | **End Coord** | |  |  |
|  |  |  |  |  | **x** | **y** |  | **x** | **y** |  |  |
| 1 | T1 | M | 5y | 30/11/2009 | 347924 | 2695220 | 25/12/2009 | 345847 | 2598932 | 589 | Tiger was captured and released back to the PTR |
| 2 | T2 | M | 2y 1m | 14/09/2012 | 355945 | 2699165 | 19/12/2012 | 266539 | 2664896 | 2294 | Tiger moved to Sagar Forest division and translocated to Satpura Tiger Reserve |
| 3 | T3 | M | 3y 4m | 25/02/2014 | 391095 | 2699447 | 23/03/2014 | 506503 | 2683263 | 642 | Tiger moved to Satna Forest division and later translocated to Sanjay Tiger Reserve |
| 4 | T4 | F | 2y | 10/11/2015 | 410111 | 2732261 | 10/01/2016 | 502519 | 2771712 | 1487 | Tiger was in Ranipur Tiger Reserve |
| 5 | T5 | M | 1y 7m | 21/07/2021 | 411941 | 2724449 | 15/10/2021 | 463693 | 2743312 | 2063 | Tiger was in Satna Forest division |

**S2 Table**: Three candidate models used for step-selection function

| **Model** | **Parameters** | **Concordance** | **Likelihood ratio test** | **Wald test** | **Score (logrank) test** |
| --- | --- | --- | --- | --- | --- |
| Model 1 | ndvi + dist_v +  sl_ + log_sl_ + cos_ta_ + strata(step_id_) | 0.605 ± 0.004 | 946.7 ***  On df 5 | 693.8 ***  On df 5 | 715.7 ***  On df 5 |
| Model 2 | ndvi * dist_v +  sl_ + log_sl_ + cos_ta_ + strata(step_id_) | 0.606 ± 0.004 | 958.3 ***  On df 6 | 703.8 ***  On df 6 | 725.3 ***  On df 6 |
| Model 3 | ndvi + dist_v +  forest_day + open_forest_day + forest_night + open_forest_night +  tod_start_:log_sl_ + tod_start_:sl_ + cos_ta_ + tod_start_:cos_ta_ +  strata(step_id_) | 0.644 ± 0.006 | 782.2 ***  On df 12 | 584.9 ***  On df 12 | 615.8 ***  On df 12 |
| Signif. codes: 0 ‘***’ 0.001 ‘**’ 0.01 ‘*’ 0.05 ‘.’ 0.1 ‘ ’ 1 | | | | | |


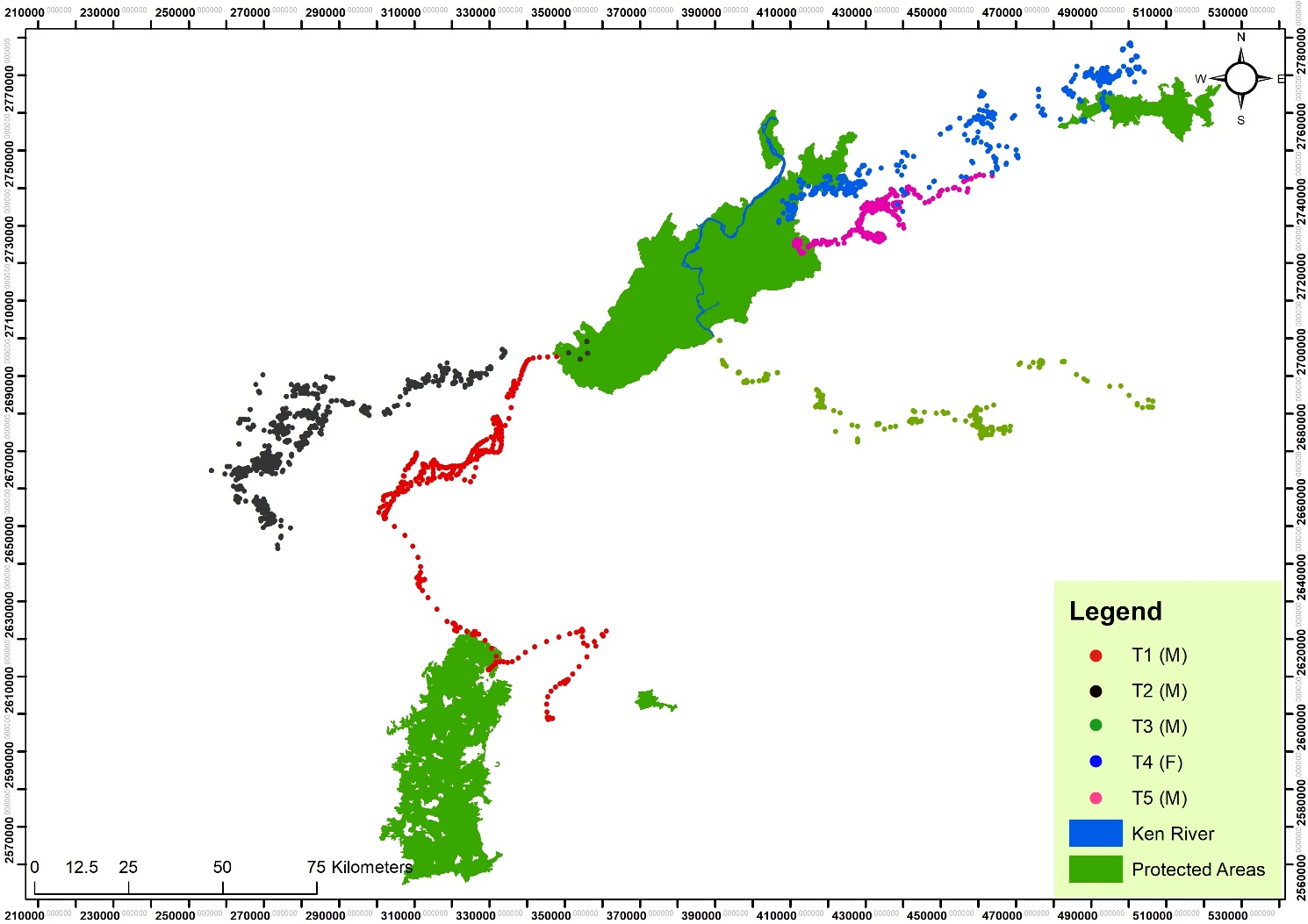


**S1 Fig**: Map showing the movement of tiger location during dispersal phase in the landscape.


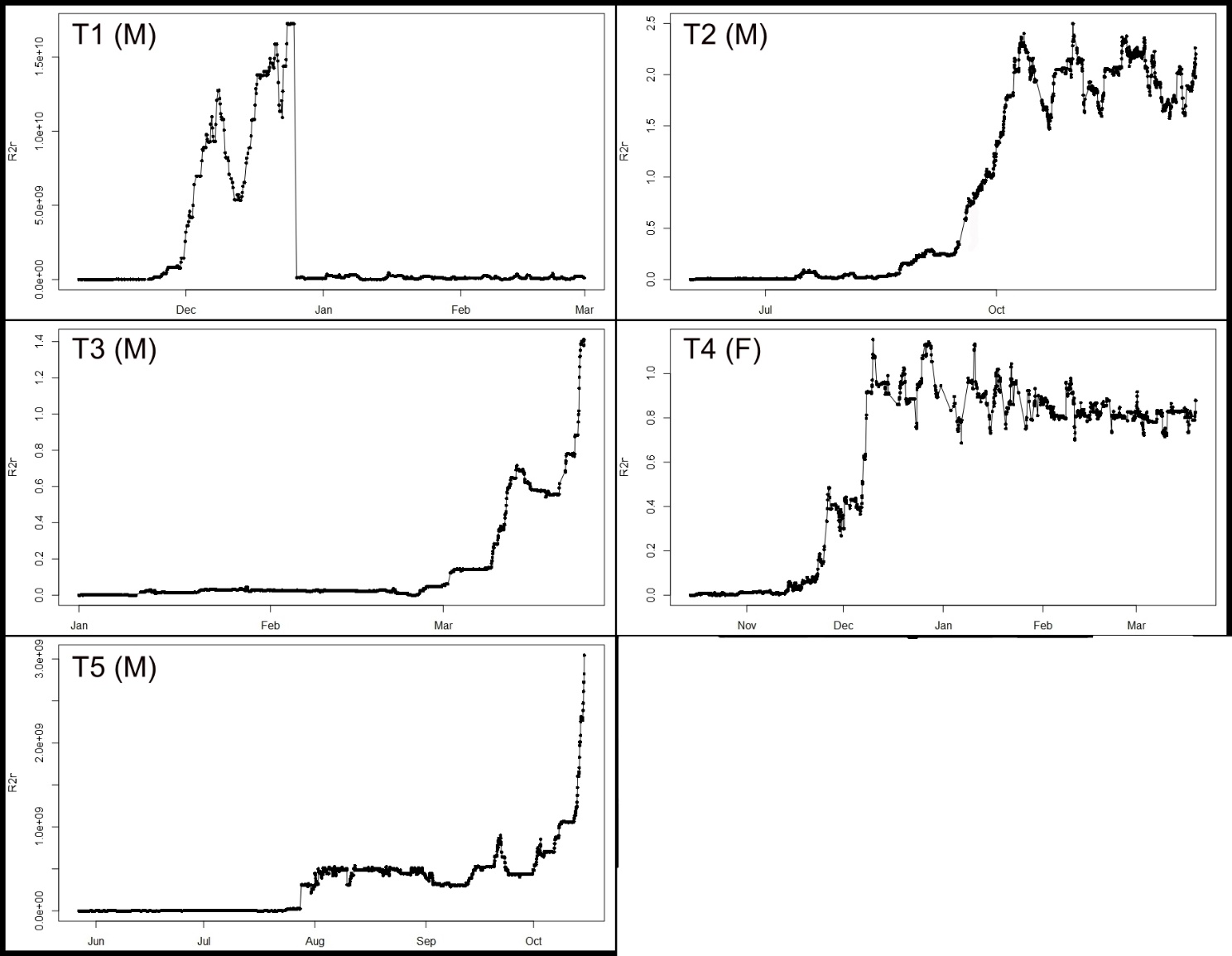


**S2 Fig**: The net square displacement (NSD) shows the dispersal time frame of five dispersed tigers in Panna landscape


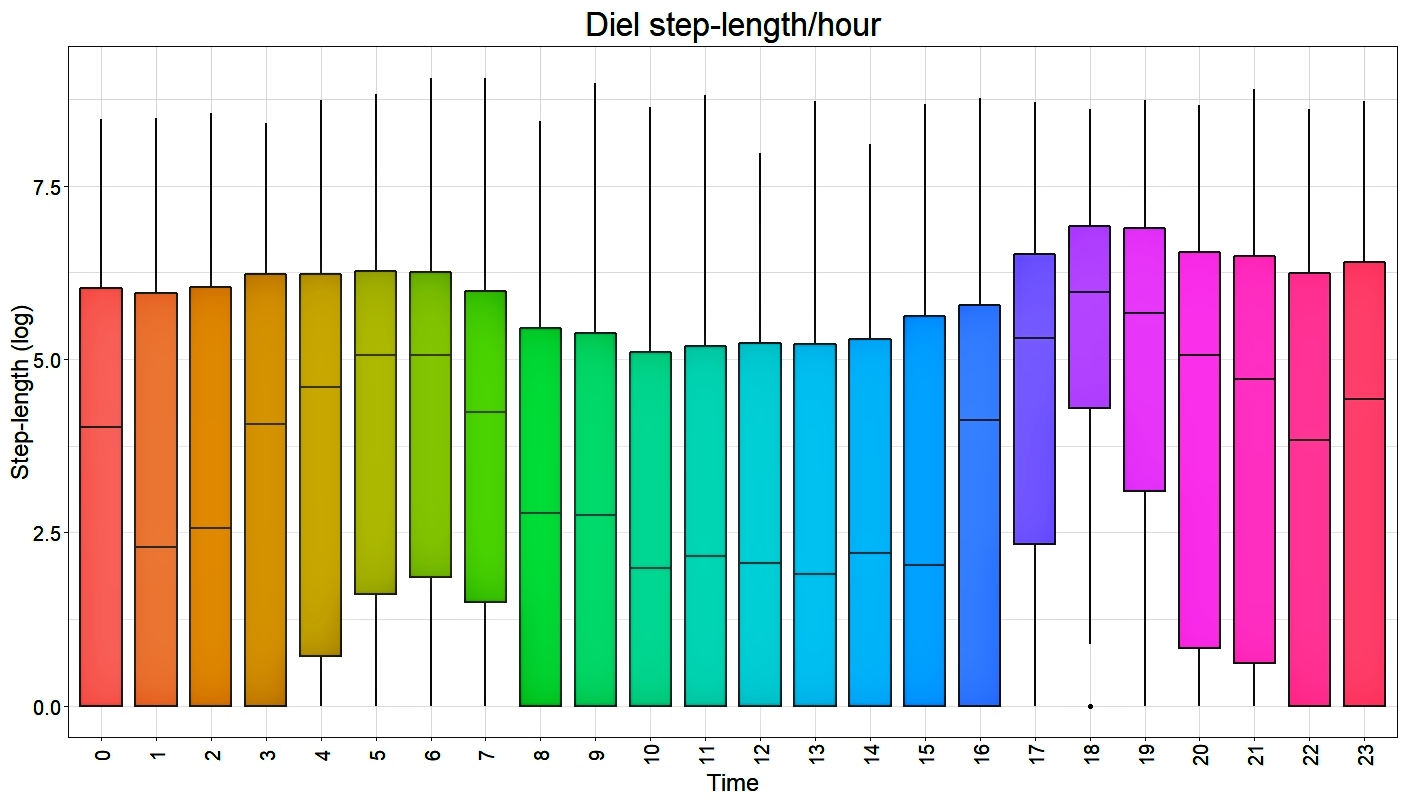


**S3 Fig:** The boxplot representing different step length (displacement) size in log-scale in different time-scale of the day.


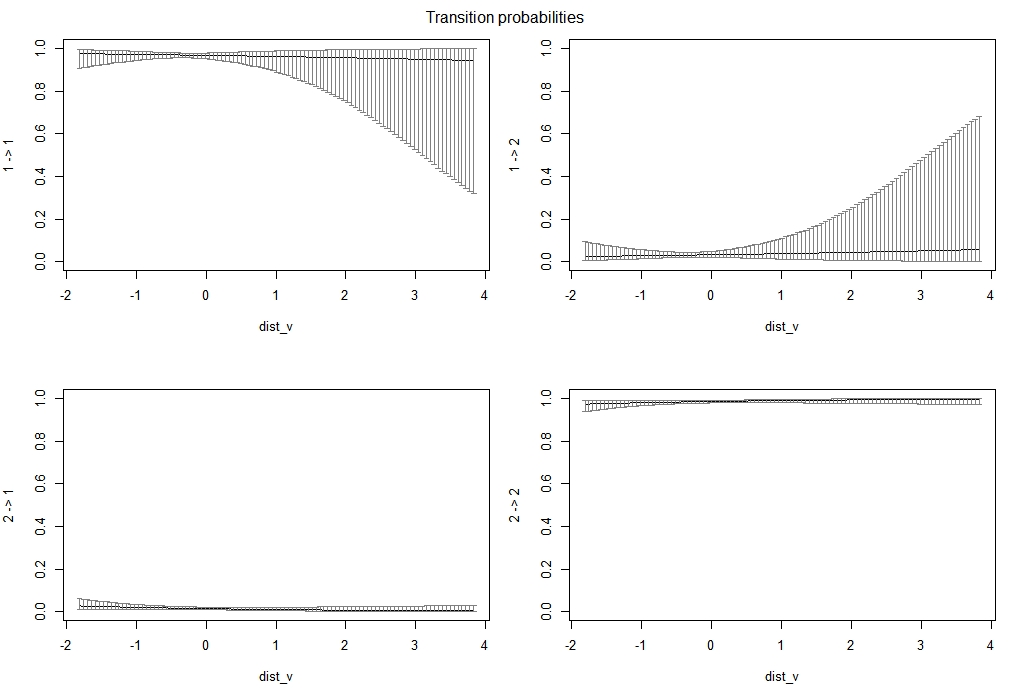


**S4 Fig**: State transition probabilities of dispersed tigers with respect to the distance from the village. The vertical lines represent the point-wise 95% confidence intervals. State 1 represents the ‘encamping’ phase, while state 2 indicates ‘travelling phase’.


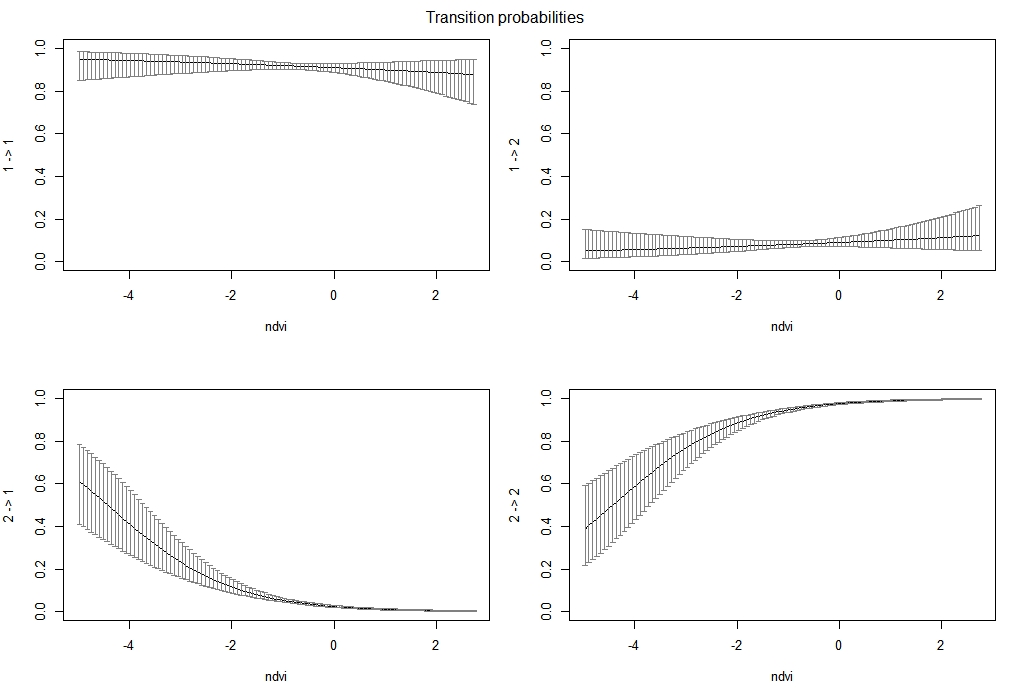


**S5 Fig**: State transition probabilities of dispersed tigers with respect to the normalized difference vegetation index. The vertical lines represent the point-wise 95% confidence intervals. State 1 represents the ‘encamping’ phase, while state 2 indicates ‘travelling phase’.


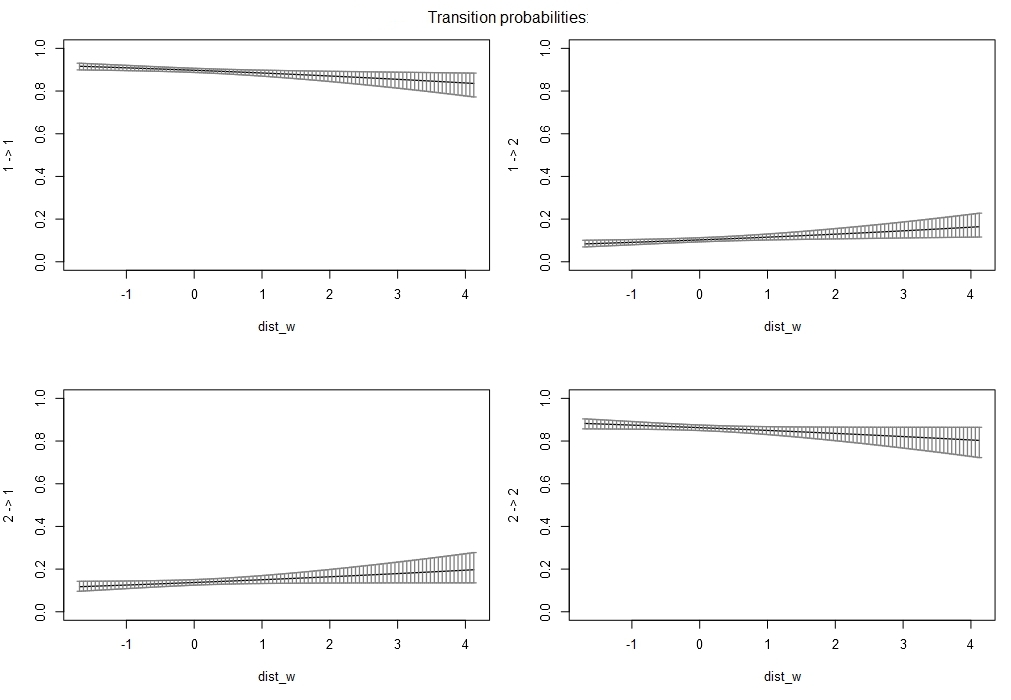


**S6 Fig**: State transition probabilities of dispersed tigers with respect to distance from the village. The vertical lines represent the point-wise 95% confidence intervals. State 1 represents the ‘encaping’ phase, while state 2 indicates ‘travelling phase’.
